# Barriers to the implementation of psychosocial interventions on acute mental health wards: an ethnographic observational study

**DOI:** 10.3389/fpsyt.2025.1501945

**Published:** 2025-02-27

**Authors:** Katherine Berry, Isobel Johnston, Paul Wilson, Gillian Haddock, Sandra Bucci, Karina Lovell, Owen Price, Adele Beinaraviciute, Gill Gilworth, Sonalia Kaur, Helen Morley, Georgia Penn, Jessica Raphael, Mica Samji, Richard J. Drake, Dawn Edge

**Affiliations:** ^1^ School of Health Sciences, Faculty of Biology, Medicine and Health, Manchester Academic Health Sciences, The University of Manchester, Manchester, United Kingdom; ^2^ Department of Research and Innovation, Greater Manchester Mental Health National Health Service (NHS) Foundation Trust, Manchester, United Kingdom

**Keywords:** ethnographic, inpatient, mental health, observations, psychosocial interventions

## Abstract

**Background:**

It is notoriously challenging to deliver psychosocial interventions on acute mental health wards. This paper presents an ethnographic observational study which captured how ward and staff processes impacted on the delivery of a psychosocial intervention called TULIPS (Talk, Understand and Listen for Inpatient Settings). Although the paper is focused on one specific intervention, the findings have implications for the delivery of other psychosocial interventions within acute mental health settings.

**Method:**

We carried out participant observation across 6 case studies wards all participating in the intervention arm of a cluster randomised controlled trial evaluating the TULIPS intervention compared to treatment as usual. Trained researchers observed ward environments, activities and social interactions taking detailed field notes which were later subject to thematic analysis.

**Results:**

Four themes were generated from field notes relating to aspects of the ward culture and staff behaviours which were barriers or facilitators to the delivery of the TULIPS intervention. Theme one highlighted how the person-centred nature of the TULIPS model was at odds with the pre-existing culture of the wards which favoured blanket rules. Theme two highlighted how staff prioritised task-oriented quantifiable activities which clashed with the emphasis the TULIPS model placed on relationship building. The third theme highlighted the presence of conflict between different groups of staff working on the ward and theme four highlighted the stressful nature of the ward environment which drove staff to seek refuge from patient facing activities including TULIPS related activities.

**Conclusions:**

In order to successfully engage with the delivery of psychosocial interventions on acute mental health wards, staff need access to supportive leadership which champions psychological interventions, as well as training, supervision and support systems which value the demanding nature of working on mental health inpatient wards.

## 1 Introduction

People with severe mental health problems benefit from psychological interventions through all stages of the care pathway. However, the delivery of psychological therapies on acute mental health wards poses particular challenges. For example, short lengths of stay may preclude the delivery of 16 sessions of therapy that people may typically receive in community settings (1). Other challenges include high levels of patient distress and risky behaviours which may prevent people from engaging in one-to-one focused therapies and consequent staff burnout and high turnover which impact on staff capacity to deliver emotionally intensive interventions (2, 3). To circumvent some of these challenges and improve patient access to psychological therapies on acute wards, we developed the Talk, Understand and Listen for InPatient Settings (TULIPS) intervention (4). The intervention was developed collaboratively by researchers, clinicians working on acute mental health wards, and patients and carers with lived experience of inpatient care.

TULIPS is a hospital ward-based intervention that aims to provide a more psychologically-informed ward environment through a stepped model of care (5), involving three levels of support, with different steps provided on the basis of patients’ needs and willingness to engage in therapy. The stepped approach helped to circumvent the aforementioned challenges associated with the inpatient environment by allowing patients to access a level of intervention that best suited their current needs. The model also included a focus on staff training and supervision and staff support to help circumvent staff burnout.

The TULIPS intervention was recently evaluated in a multi-centre cluster randomised controlled trial involving 34 wards in the UK, randomised to receive TULIPS or usual care, the results of which will be reported elsewhere. The evaluation included a nested process evaluation to assess fidelity to the intervention and barriers and facilitators to model delivery through qualitative research. This paper reports an ethnographic observational study which was part of the process evaluation Our fidelity assessment highlighted variation in how well intervention was delivered across different wards but in general there was lower fidelity for aspects of the intervention which necessitated nursing staff to engage in the delivery of psychological interventions (6). Our interview study and observational study sought to understand the barriers and facilitators that explained this variation in practice and issues with delivery. The observational study was particularly focused on ward cultures and ward staff behaviours given that the delivery of the intervention was so reliant on the participation of all members of the multidisciplinary team. Ethnographic observations are highly compatible with the goals of process evaluation, enabling an understanding of behaviour in context and circumventing biases inherent in interviews (7). Observational methods are a well-established approach to understand processes in health care (8) and within acute mental health inpatient care settings have been shown to reveal complexities of the job roles not captured by other research methods (9).

The overall aim our observational study was to understand the factors that inhibited or enhanced the delivery of the TULIPS intervention. Although the paper is focused on one specific intervention, the findings have implications for the delivery of other psychosocial interventions within acute mental health settings.

## 2 Methods

### 2.1 TULIPS model

The intervention was delivered by a Health Care Council Professions registered clinical or counselling psychologist who was based on each ward 2 ½ days per week. Prior to commencing the intervention, the psychologist provided training to ward staff in psychological ways of working including the role of interpersonal trauma and social adversity in the development of mental health problems and structured approaches to addressing mental health problems commonly experienced by patients on the ward (e.g. psychosis, anxiety, low mood, self-harm, emotional dysregulation and anger). The purpose of training staff was to enhance staff knowledge and confidence in engaging in psychological work with patients. As outlined in the introduction, the TULIPS model was designed as a stepped model of care. At Step one, patients receive a psychological formulation developed by a psychologist in conjunction with the patient or members of the ward team. A formulation provides a framework for bringing together biological, societal, cultural and psychological factors that might be responsible for the development and maintenance of problems and thus facilitate planning and implementing the most appropriate intervention (10). Discussions arising from the formulation at Step one determine whether or not the patient would benefit from being offered a Step two or three intervention. At Step two, staff are trained and supervised by a psychologist to deliver guided self-help material of psychological interventions targeting key problem areas for patients (e.g. anxiety management, behavioural activation for low mood, coping with symptoms of psychosis, minimising self-harm behaviours and suicidality). At Step three, patients perceived to have needs that cannot be met at Step 2 and who want to engage in psychological therapy are offered up to 16, one-to-one therapy sessions with the psychologist. In addition to delivering the stepped model of care, the psychologist also delivers fortnightly group-based, staff reflective practice sessions of one-hour duration and fortnightly team formulation sessions. The purpose of these sessions was to support staff members’ capacity for engaging in psychological work with patients.

### 2.2 Observation settings and time points

Ethnographic observations were conducted on four male and two female acute mental health wards randomised to the intervention arm of the TULIPS trial. These wards varied by location (three rural and three urban). Observations were completed on each ward following the start of the intervention. A total of 414 hours and 45 minutes of observations were completed across 64 days, divided across three time-points on each ward over a nine-month window. One time-point on one ward was not completed due to COVID-19 restrictions in place at the time. Each time-point included an average of four observation days (ranging from two to five days). The length of each observation day was 6 hours and 37 minutes on average (excluding two outliers where observations days were terminated early due to high levels of violence on the ward and the researcher being advised by staff to leave) and ranged from 5 hours and 45 minutes to 8 hours and 15 minutes.

### 2.3 Research team

Observations were conducted primarily by three postgraduate researchers (including authors GG and IJ) trained by DE in ethnographic observation and field note taking. Researchers refrained from engaging with the delivery of care and passively observed meetings and interactions. The researcher’s presence and role was not concealed from participants (11).

### 2.4 Ethical issues

Ethical approval for this study was provided by the Greater Manchester East Research Ethics Committee (IRAS ID: 264686). Ward manager permission was obtained before researchers attended wards. Informed consent from staff and patients was only sought when observing private meetings (such as ward rounds, formulations, supervision). When staff declined to participate, they were omitted from field notes and the meeting was still observed. However, when patients declined to participate, the observer did not attend the meeting. When completing observations in communal areas, posters were displayed making staff and patients aware when and where observations were being completed.

### 2.5 Participants and data collected

Researchers observed ward environments, activities and interactions between those present (i.e. staff and patients). Observations were completed at varying times between 7am and 8pm to ensure core ward hours were observed. Efforts were made to attend a variety of regular meetings and activities (e.g. ward rounds, coffee mornings, handovers, and formulation sessions).

A total of 176 people were approached to consent to observations of private meetings (63 patients; 113 staff), with 167 providing informed consent (55 Patients; 112 staff). Eight patients who were approached declined participation; five due to feeling there would be too many people present in the meeting and three patients gave no reason). One staff member declined participation due to feeling uncomfortable being observed.

Detailed field notes focused on interactions between ward staff and patients present during the observation period, the ward environment and practices, and the delivery of the intervention (12). For example, the researchers observed interactions between staff members with each other and between staff and patients. They noted the content of what was said as well as reflections on non-verbal communication within the situation. The researchers also positioned themselves at various locations within the ward and noted the level and type of activity that took place and the mood of the ward in terms of level of staff and patient activity, noise levels and sense of threat. The researchers further noted any interactions that patients or staff instigated with themselves. The field notes were typed up from handwritten notes as soon as possible following each observation period.

### 2.6 Analysis

Observation notes were analysed using thematic analysis informed by Braun and Clarke’s six-step approach to reflexive thematic analysis (13, 14). Three members of the research team (IJ, MS, AB) were involved in the analysis. The purpose of involving multiple researchers in the analysis was not to demonstrate reliability of coding but to help manage the volume of work and to bring different perspectives to the analysis. Observation notes were organised into observation time-points by the IJ and data was managed and coded using the NVivo software.

First all researchers involved in the analysis familiarised themselves with the data by reading all notes. Two time-points were then selected at random and all researchers inductively coded this data following a second reading. After this initial coding, the researchers met to sense check their individual coding and all codes from the two time points were collated into a codebook. The purpose of the codebook was not to help ensure reliability of coding but to facilitate the process of managing a large dataset with multiple coders. Going forward, the data was divided between the researchers and each person coded independently into the codebook.

All data felt to be incongruent with the codebook was labelled with ‘other’, reviewed and discussed during regular coding meetings. The codebook was designed to be adaptive and iterative so new codes could be added, and existing codes could be adapted, merged or relabelled as necessary. This process led to five iterations of the codebook, with the number of codes increasing from 57, in the first iteration, to 97 codes, in the final codebook (see **Supplementary Material**). These meetings also facilitated discussions of how codes could be group into possible themes representing recurring ideas and reflections of influences on the data analysis (see reflexivity section).

After coding, themes discussed throughout analysis were labelled and expanded upon and additional themes were developed. This process of theme generation was aided by paper notes containing code names and descriptions, and with reference to the raw data in NVivo. Early themes were regularly discussed during supervision sessions with DE, PW and KB and presented to wider research team who gave feedback on the coherence and organisation of themes, as well as theme labels. Early themes were also presented to the TULIPS patient and carer lived experience group, comprising four members with lived experience of mental health services as patients or carers. In this meeting, raw data from each theme was presented without interpretation. Group members were encouraged to provide their interpretation. Detailed notes were taken and compared to researchers’ interpretation of themes, validating theme summaries or increasing the richness of understandings where additional insights were provided. For example, group members talked at length about patient experiences of feeling neglected by staff on the wards, wanting to be acknowledged and heard, but staff often appear too busy to engage.

### 2.7 Reflexivity

All authors involved in the collection and analysis of data were female and employed researchers on the TULIPS project. Data analysis was completed by IJ, MS and AB. Authors DE, KB and PW supervised data collection and provided feedback on themes interpretation throughout the analysis process. Authors AB, DE, GG, IJ, SK, GP, MS and PW had not worked on acute mental health wards prior to this study. However, IJ, SK and MS all had experience working in other inpatient mental health settings. KB had previously worked on mental health wards as a psychologist, but the other supervisors had not. Our individual backgrounds influenced the behaviour and interactions we attended to and the lens through which interpreted observations and resultant field notes. For example, those who had previously worked within acute mental health settings may have better appreciated the challenges from the staff members’ perspectives. All members of the team were also committed to increasing access to psychological therapies and reducing the medicalisation of psychological distress, which meant increased value may have been given to psychological approaches. The variation in researchers’ exposure to acute mental health wards, and mental health settings more broadly, was beneficial in providing context to field notes (in the case of those prior exposure to acute mental health wards) and facilitating more novel interpretations of the data (in the case of those without exposure to this setting).

## 3 Results

We generated four themes related to aspects of the ward culture and staff behaviours which were a barrier or facilitator to the delivery of the TULIPS intervention and impacted on ward staff engagement with the ethos and components of the TULIPS intervention. Theme one highlighted how the person-centred nature of the TULIPS model was at odds with the pre-existing culture of the wards which operated on the basis of inflexible rules governing all aspects of patient care. Relatedly, theme two highlighted how staff prioritised task-oriented quantifiable activities which clashed with the emphasis the TULIPS model placed on relationship building with patients and meant that the TULIPS work was not prioritised. The third theme highlighted how conflict between different groups of staff working on the ward, which centred on concerns about unequal workloads may have driven staff to focus on task-orientated quantitative activities at the expense of TULIPS activities, or not engage with TULIPS activities due to perceiving the intervention as a criticism of existing practice. Theme four highlighted how the stressful nature of the ward environment also drove staff to seek refuge from patient facing activities, which were the foundation of the TULIPS model.

The themes are described in more detail below. They are supported by verbatim extracts from field notes which are presented alongside anonymised ward identifiers and the associated time-point from which extracts originated (e.g. Ward 3 Time-point 3). Where relevant, we also highlight changes in practice that were observed following the implementation of the TULIPS model.

### 3.1 Conflicting cultures

The ethos of the TULIPS model was person-centred in that it aimed to consider each person’s needs and circumstances to inform decision making about care. For example, during an observation of a ward round where the typical blanket rule of suspending leave from the ward was being discussed as a way of managing a patient’s behaviour, the TULIPS psychologist was observed advocating for a more flexible and less punitive approach regarding leave.

*Staff begin discussing the next service user. They have also had their leave suspended due to drinking whilst in the community. There is a discussion that he was honest about it and opened up. Psychologist advises “I feel as though we need to be a bit flexible here, we’ve taken a lot away from him and we need to give something back”* Ward 6 TP2

However, the person-centred ethos of the TULIPS approach often contrasted with the existing ethos of the wards, which was generally heavily rule focused with little flexibility or negotiation with patients about what behaviours or actions were permissible. For example, ward staff were often observed voicing and enforcing ward rules around actions that were, or were not, permitted. This contrast in ethos was a significant barrier to implementing the TULIPS intervention as it required a consequent need to shift fundamental ways in which staff operated the ward. As highlighted in the extracts below, ward rules not only dictated the acceptability of patients’ actions, but also the precise time periods within which actions and tasks needed to be performed, highlighting the minutia and coercive nature of the rules and the extent to which ward staff dictated life on the wards.

*Patient H comes to the hatch and complains to nurse G that he ordered a McDonalds, and no one would go to the ward door to collect it for him. Nurse G response: “It’s Monday today, no takeaways on Mondays”, Patient H “but it was at the door”, Nurse G “it doesn’t matter we won’t accept it”* Ward 5 TP3

*Patient S says that he thought that they were going to try to sort out later breakfast because he and another patient like to lie in and often miss breakfast altogether. M says, “so you want us to change the breakfast time just for you and X?” His speaking tone is a bit incredulous when he says this. M adds, “don’t forget that this is a hospital, even if you were staying at a hotel breakfast would only be a certain time and if you went down too late after breakfast had finished you wouldn’t get anything”.* Ward 3 TP3

As shown in the following example, the rigid prioritising of obeying rules and following standard processes which took precedence over individual patients’ needs appeared to be a frequent trigger for or a maintaining factor in a patient’s distress.

*“The nurse opened the nursing office door, and the patient came in behind her to get her possessions out of her draw since she was packing to leave. However, the nurse said she had to leave them in there until she was about to actually go. Again, this restriction of getting her own possessions when “she was no longer going to be a patient” meant that she began getting frustrated again and shouting about the situation”*. Ward 1 TP2

Staff also frequently used coercion and an authoritative stance rather than the therapeutic relationship to ensure that patients behaved in line with the rules. As seen in the examples below, the perceived need to enforce rules as paramount resulted in a non-therapeutic approach that was at odds with the person-centred TULIPS model.

*Nursing Assistant enters and can be heard sternly telling patient 16 that he “needs to stop shouting, you’re disturbing everyone on the corridor, if you need something from us you can just come and ask us but if you keep shouting, we might have to think about moving you back to the de-stimulation lounge”.* Ward 6 TP1

*When patient doesn’t appear to accept the explanation about her requests for more medication, the staff nurse changes her tone to become more abrupt and her tone as if she is speaking to a child. After she has given the patient a cigarette the staff nurse closes the door in the patient’s face.* Ward 2 TP1

Through training, team formulation, reflective practice and supervision, the TULIPS intervention aimed to help staff understand the drivers behind patients’ behaviours, validate distress and avoid responding in punitive ways that may ultimately escalate conflict and aggression. This alternative way of responding is illustrated in the following example observed after implementation of the TULIPS model. In contrast to the above observations, and in keeping with the person-centred approach advocated by the TULIPS model, the ward nurse was able to respond sensitively to the patient’s distress and perceived need for medication and in turn support the patient to find alternative ways of coping with her distress.

*Patient J is outside the nurses’ office, she appears mildly agitated. A staff nurse comes out of the office to speak with her. J wants PRN medication, the nurse explains patiently to her that it is too close to her last dose for her to have more medication. The nurse has to repeat this multiple times as J remains agitated and does not seem to be listening. The nurse then suggests maybe they could go somewhere together to chat and asks J if she has any ideas about what she could do to distract herself.* Ward 2 TP2

### 3.2 Task-focused nursing role

Related to the rigid focus on the enforcement of rules was the precedence that ward staff gave to tasks that were quantifiable and directly measured over engaging patients in conversation and relationship building which were fundamental to the TULIPS model. For example, the majority of qualified nursing staff time was spent in the office engaging in tasks, such as writing up notes or completing care plans. Pointedly, in the following extracts staff labelled these office-based activities as ‘work’, implying that direct patient contact which the TULIPS model sought to promote was not.

*The nurse is now stood in the doorway listening to the patient’s frustration about medication. After a few minutes the nurse told the patient she was closing the door because she “needed to get work done”. The patient shouted about this, saying not to close the door on her again.* Ward 1 TP1

*The patient follows them in making the staff nurse jump as she didn’t realise that the patient was behind her. Everybody laughs including the patient who persistently tries to hug the staff nurse who again pushes her gently away. The staff nurse explains to the patient again that she needs to get on with her work and will see her later. The staff nurse closes the office door with the patient outside* Ward 2 TP1

One of the key activities that brought nurses out of the office and onto the wards was administering medication. Arguably, this could have been an opportunity to build rapport with patients whilst engaging in a well-established activity on the ward that was an endorsed and acceptable use of the nurses’ time. However, as illustrated in the following example, this activity was often completed with minimal staff and patient interaction and with no attempt to invite patient conversation, suggesting that other factors presented in subsequent themes may also be at play in mitigating against the relationship building activities that were the foundation of the TULIPS model.

*At the meds clinic there is no small talk and the nurse is looking stressed due to having to do so many tasks at once. Instead, the nurse is focused on getting through the patients as quickly as possible, with little small talk.* Ward 1 TP2

Nursing assistants were more present on the wards than nurses and in theory had more time available for relationship building with patients, but most of the activity that they too engaged in was task-focused and, like the nurses’ activities, involved minimal communication with patients. For example, frequently observed nurse assistant activities included walking around with a clipboard carrying out observations, letting people in and out of the ward, and supporting nurses to administer medication. The following extracts are examples which demonstrates lots of staff presence but minimal staff and patient interactions.

*Nursing assistant 18 is doing observation checks but without interacting with patients, or only having short brief check ins. Lots of nursing assistants are present on the ward but are all doing their own things and not really engaging with one and other of the patients.* Ward 6 TP2

*Lunch time being facilitated in the day room by nursing assistant D and nursing assistant E, they are standing around the food trolley by the entrance to the day room while 8 or so patients sit spread across the dining room eating lunch. Patients aren’t talking to one another, and nursing assistant D and nursing assistant Es engagement with patients is minimal and task focused (e.g. “which dinner did you order X?”).* Ward 4 TP2

The observers reflected on the reasons for the staff members’ prioritisation of tasks and, in particular, whether or not withdrawal from patient interactions was enabling staff to avoid engaging in more emotionally intensive work. In support of this hypothesis., there were overt examples of staff ignoring patients’ needs and emotional draining work. For example, the following interaction during handover suggests that staff may be prioritising medication compliance over therapeutic interactions to address distressing symptoms.

*Charge Nurse M is delivering the handover to the day shift. They remark Patient P isn’t very compliant with medication because he is suspicious. We had to get all the staff to enforce it. He had most of it, the most important ones and hopefully the more meds he has the more compliant he is. Can you make sure he has medication tonight so he sleeps and its easier on us.* Ward 5 TP1

However, it was also noted that the tasks which staff focused their attention on were those that carried sanctions for not completing them, such as care plans, observations and medication rounds. TULIPS intervention sessions (including one-to-one sessions that nurses were expected to deliver with patients and supervision or formulation sessions delivered by psychologists that nurses were expected to attend) were not prioritised, as there was no immediate consequence for staff not engaging. The priority given to auditable tasks was most evident when unforeseen events happened on the wards, such as staff sickness and serious incidents. As seen in the extracts below, when ward tasks needed to be prioritised, it was often the TULIPS activities that were cancelled.

*The depression group that was meant to run today has also been cancelled, as there are no staff to run it. Due to unforeseen events happening on the ward frequently, trying to schedule activities or events was difficult and led to disappointment for patients when they were cancelled.* Ward 1 TP2

*The Psychology group is meant to be happening now with the patients; however, it hasn’t been announced and I have not seen anyone come onto the ward to do it. I asked nursing assistant V if he thinks it will happen and he said “No, it never happens. They usually need a nursing assistant to sit in but they can’t because they are understaffed*.” Ward 4 TP3

A focus on developing relationships with patients to help understand their needs and prevent frequent escalations of distress was a key component of the TULIPS model, but the usual ward priority was given to office-based or practical tasks leaving limited time for relationship building. Relationship building is a necessary element to the TULIPS approach to encourage patients to engage in psychosocial interactions with staff. Without this fundamental platform, patients may have felt reluctant to enter one-to-one interventions sessions with nurses. There were, however, examples post implementation of the TULIPS model of staff using task-orientated activities to develop relationships with patients.

*A staff nurse is in the clinic room with a student nurse ‘C’. The first few ladies who appear for medication appear very sleepy. When another patient approaches for her medication nurse C asks how she’s feeling today. This patient asks the student nurse a number of questions about her possible discharge. Both staff take their time to answer the patient’s questions in full and offer reassurance.* Ward 2 TP2

However, more commonly, offers of support were reactive in response to patients’ expressions of frustration or upset rather than proactive. In these instances, staff engaged sincerely, providing quality time to talk, evoking a sense of care for, and compassion towards, patients. Nonetheless, the focus on risk management as opposed to using strategies to prevent risk escalation is at odds with the ethos of the TULIPS model and provides a further example of a clash between the TULIPS model and existing ward culture.

*Patient T knocks on the door. The bank nursing assistant opens the office door (from the outside), she says to patient T “can you just go over there” in an abrupt tone. Patient T comes back and bangs on the office door loudly, when there is no reply she sits on the floor outside the office crying. Nursing assistant A comes out of the office and asks T “why are you crying?” (her tone is sympathetic). Patient T replies: “because I need to ring my Mum to tell her where I am, she’s driving up to see me”. Nursing assistant D comes over. Nursing assistants A and D spent a few minutes talking to patient T telling her that she has already used the phone a couple of times this morning to speak to her Mum. Nursing assistant D then suggests that maybe she could go out into the garden to get some fresh air.* Ward 2 TP3

### 3.3 Staff conflict about workloads

Conflict between staff members was also evident on the wards and most frequently centred around perceptions that the workload was unevenly distributed and a resulting sense of unfairness. As highlighted in the following extracts, the conflict often focused on groups of staff within specific roles, such as senior management, qualified nurses, nursing assistants or medical staff and related to lack of appreciation of other professionals’ roles. It was often heightened during busy periods on the ward when staff were feeling under pressure and operating in threat mode with an ‘us and them’ mentality.

*Nurse A began discussing a new admission to which Nurse C explained they were admitted at eight, but the doctor didn’t arrive till three and then rolled their eyes, to which the other staff laughed and made jokes “maybe the traffic was bad”, “did he stop for a kebab on the way”.* Ward 5 TP2

*The nurses also expressed displeasure at the “high-ups” who they felt didn’t understand the role of tobacco on the wards in helping to keep patient tensions at bay. Patients were given handouts about the ward being tobacco free, and that their cigarettes would be thrown away on Sunday if they brought any on to the ward. Nurses said that it was another thing being taken away and “Just wait until next week to see how stressful it will be here”.* Ward 1 TP1

*I asked nursing assistant F what he was doing as I’d seen him walking up and down the corridors and he advised “I’m a busy man, doing lots instead of hiding in the office” alluding to the fact the nursing staff had spent most of the day in the ward office.* Ward 5 TP2

Interprofessional conflict may have impacted on the delivery of the TULIPS intervention in two main ways. First, the fear of implicit or explicit criticism from colleagues about not pulling one’s weight may have led some staff to prioritise quantifiable and measurable tasks described in the previous theme, meaning that relationship building with patients and other TULIPS intervention activities that were not measured were not prioritised. Second, staff may have perceived the clash between the TULIPS model and existing ward culture as a criticism of their existing practice, which may have reduced their buy-in and likelihood of engaging. This perceived criticism may have meant ward staff were not receptive to the psychologist’s advice regarding patient care or engaging in team formulation, reflective practice or supervision sessions delivered by psychologists. There was, however, no overt evidence from the observations that the staff resented the TULIPS psychologist’s advice. On the contrary, as shown in the following example, there was evidence to suggest that staff valued the input, facilitated by the psychologist’s empathetic approach to advising staff.

*Psychologist can be seen chatting to nurse 1. Nurse 1 is seeking support regarding a particular patient who is due to be discharged but nurse 1 is anxious that they are not quite ready. They discuss his recent behaviour and psychologist uses encouragers (mm, yes, hmm) and smiles reassuringly at nurse 1. She asks questions “that sounds really difficult, I can imagine” and “what do you mean by that?”. They continue what seems to be an informal formulation talking through nurse 1’s understanding of the patient’s behaviour and his concerns that without the support of the ward the patient will struggle.* Ward 6 TP2

### 3.4 Staff seeking respite

The wards were busy and pressured environments aggravated by the large number of tasks to complete, the underlying conflict about workloads and unpredictable changes in resource. Staff were often observed in the nursing office with colleagues, and as seen in the following examples, this was a place where staff sought support from each other and there was a lightened mood.

*Charge nurse 1, staff nurse 1, staff nurse 2 and activity co-ordinator can be heard laughing and joking in the ward office loudly although what they are saying is inaudible.* Ward 6 TP2

*A nursing assistant comes back onto the ward following a break bringing a four pack of energy drinks, there is some light-hearted banter in the office about the staff really needing more than just some caffeine.* Ward 2 TP2

Unlike other members of the multi-disciplinary team, nurses did not have their own space off the ward to gain respite, meaning that the nursing office served this function. However, as seen in the following example, the nursing office door was a pinch point for conflict, with multiple patients observed waiting outside, repeatedly knocking, and becoming irate with staff who answered.

*A patient was angrily banging on the nurse’s office door to try and get their attention. I don’t know what had happened but the one member of staff in the room was ignoring the banging. Another patient elsewhere was crying in the corridor as it was “too noisy everywhere”. She was stood against the wall mildly panicking and holding herself. Someone else was sat in the corridor telling everyone they were dying.* Ward 1 TP1

The fact that the ward itself was so pressured and the nursing office acted as a refuge for staff meant that staff retreated into the office rather than engaging with patients in relationship building or preventing the escalation of incidents on the ward. As seen in the following extracts, there were often no staff present on the ward as all staff were in the office.

*There are no staff visible on the ward, all the nursing assistants on shift and nurses are in the nurse’s base.*Ward 3 TP3

*Staff were mainly in the ward office and there was a general lack of presence in the day room where most patients were now sitting watching TV or having hot drinks.*Ward 5 TP2

## 4 Discussion

The purpose of this ethnographic observational study was to understand the factors that inhibited or enhanced engagement with the TULIPS intervention and may similarly impact on the implementation of other hospital ward-based psychosocial interventions. The findings suggest that the person-centred nature of the TULIPS intervention was at odds with the uniform rules and processes that wards typically operate within. The person-centred model with staff and patient relationship building at its core also ran counter to the ward staff prioritisation of task-focused quantitative activities, which required less emotional investments and carried sanctions if they were not completed. The focus on individual formulations of patients’ needs within the TULIPS model helped to challenge the use of blanket rules within the ward and emphasised the importance of the relationship building aspect of the nursing role. However, the stressful nature of the ward environment and the nursing role triggered intergroup conflicts related to perceived disparities in workloads, which may have resulted in staff prioritising measurable activities such as administrative work and perceiving the introduction of the TULIPS intervention as a criticism of their existing practice. Psychologists delivering the TULIPS model avoided possible conflict regarding differences in workloads by investing time in developing good relationships with other ward staff. The stressful nature of the ward environment and prioritisation of administrative duties also drove staff to retreat to the office rather than being present on the wards which hindered relationship building activities with patients in turn fuelled patient distress and the high levels of emotions on the ward.

It is well documented that inpatient settings are governed by rules that dictate how patients should and should not behave (15, 16) and that these rules hinder the implementation of psychological interventions which are more person-centred (16). Ward rules often relate to restrictive practices, which are practices implemented to reduce risk but impede a person’s freedom, rights and daily activities (17). Although the containment offered by predictable rules and consequences can enhance feelings of certainty and safety for some patients (18), restrictive practices and inflexible rules are disempowering, increase patient stress and frustration and can inadvertently cause physical and psychological harm to both staff and patients (17, 18). Formulation, which was one of the fundamental elements of the TULIPS model, summarises a person’s unique life history and the impact of experiences on current beliefs, ways of coping and relationships, and as such provides a means of understanding a person’s unique needs and developing strategies to support the person without needing to resort to restrictive interventions. The Safewards model, an evidenced-based approach for therapeutically reducing conflict on acute inpatient wards, also advocates reducing patient powerlessness in response to rules through staff and service users collaboratively identifying and agreeing to a list of standards for the ward (19). These standards should then be displayed on the ward and included in the admission process. Our observations and previous observational research within inpatient settings further suggests that nurses differ in how they implement ward rules meaning that rules which are deemed important for patient safety do not have to be enforced in a punitive and authoritarian manner. For example, some nurses may use a more gentle tone or even humour to make situations more acceptable and person-centred for patients (9). It is also important to note that some of the rules that were observed were not directly related to patient safety (e.g. mealtimes, purchasing of takeaways) and were therefore coercive. It was unlikely that these rules were related to general hospital policy, but they were ward specific rules arising from paternalistic attitudes towards patient care. Previous research has suggested that these ward rules are often implicit with patients only becoming aware of them when they face the consequences for transgressing them which can contribute to the stress and sense of powerlessness associated with an inpatient stay (20).

The observation that nurses prioritised task-focused quantifiable activities at the expense of relationship building or TULIPS intervention sessions was corroborated by findings from our fidelity study, which measured the degree to which different aspects of the model were delivered (6). As highlighted in the introduction, our fidelity data suggested that nurse-led TULIPS interventions were particularly poorly implemented (6). The small amount of time that nurses are able to spend listening and talking to patients due to the high level of administrative work is a well-established finding (2, 21) and has been shown to impact on nurses’ job satisfaction (21). In this respect, systems of bureaucracy limit actual staff and patient engagement, with attention to the perceived importance of ‘paperwork’ justifying the time staff spend away from patients.

Other professional groups and academic researchers often criticise nurses for neglecting therapeutic activities with patients (9), but to do so assumes that nurses are to blame. The nurse’s role is multifaceted with numerous competing demands that are essential for maintaining patient safety which in itself can be emotionally draining (9). Mental health inpatient nurses are also frequently subjected to physical assaults and threats of violence or other verbal abuse including racism and sexual harassment (22, 23). They frequently witness acts of self-harm and hear graphic accounts of suicide attempts or childhood abuse (24, 25). Despite the potentially traumatising nature of this work, staff are often not well supported through reliable and supportive supervision structure and face barriers in accessing staff support systems that are available (26, 27. Within this context, our observations of instances where nurses seem to ignore patient distress is not surprising and may serve to help nurses function within their role. As acute mental health wards are risk averse environments where staff blame and accountability are high, it is also not surprising that the focus of nurses’ time and attention easily gets drawn into processes that are mandatory and auditable (2). Research further suggests that in times of stress or fatigue, health care professionals can revert to engaging in non-reflective routine behaviours which adversely impacts on the delivery of novel healthcare interventions (28). Our data and previous observational research does, however, suggest that nurses can use routine activities such as medication rounds to skilfully engage patients in therapeutic interactions (9).

Results from our complementary interview study (Berry et al., in submission) and previous research (2, 28) suggests that staff engagement with novel interventions is enhanced when team leaders prioritise staff engagement in the activity and team leaders who encourage open communicate with and feedback from staff about changes in procedures (28). Previous research also highlights that ward staff are more likely to engage in delivering psychological interventions when they feel accountable for the activity which can be achieved through auditing the therapy that is delivered against ward level targets (2). Similarly, research suggests that healthcare professionals are more likely to engage in novel practices if the engagement has clear consequences (28). It is, however, equally important that staff feel skilled and equipped to deliver interventions through adequate training, and supervision structures, including opportunities to observe experts and practice skills *in vivo* (28).

There were many observed instances of staff camaraderie which often centred around the staff office. A strong team culture and mutuality amongst nursing teams has been attributed to the fact that nurses are on the front line in a risky environment and rely on each other in dangerous situations that other health professionals do not have to routinely manage (9). There was, however, evidence of conflict between different groups of staff, including nurses and nursing assistants, and nurses and other members of the multidisciplinary team. Intergroup conflict is a common phenomenon in stressful environments (29) and has previously been documented amongst staff working in mental health care settings (30). In terms of conflict, previous research also highlights how the implementation of new interventions by research teams can be perceived as criticism of existing practice, which results in limited staff engagement (2). More specifically, previous research has found that psychologists can be seen to be unwelcome ‘experts’ by other members of the multidisciplinary team who do not appreciate the realities of the nurse’s role (31, 32). In order to overcome this potential perception, we deliberately ensured that psychologists delivering the TULIPS intervention were ward-based and invested time in developing relationships with the team. We also ensured that the intervention was developed in collaboration with staff with current experience of working on acute mental health wards including managers, staff nurses and nursing assistants (6).

Our observations suggested that the staff office was an important sanctuary where staff could focus on administrative tasks and socialise with each other. This space was hypothesised to be particularly important for nurses, as unlike other members of the multidisciplinary team, the nurses did not have a professional space outside of the ward environment. Although the sanctuary of the staff office was important for staff wellbeing, consistent with previous observational research on mental health wards, the office door was a central point for staff and patient conflict (33). The office was a powerful symbol of staff power as patients were seldom permitted to enter the office and the door is a physical barrier shutting them out of a place where important decisions are made about their care (33). Previous research suggests that open plan nursing stations or even leaving the office door open can allow freer contact between staff and patients (33), but in making such changes it is important for nursing staff to have alternative space off the ward to recoup and support each other.

### 4.1 Strengths and limitations

The ethnographic observational study permitted unique insights into the nurse’s role and the ward culture that were not elicited through our complementary interview or fidelity studies. It is possible that the staff we interviewed withheld information due to social desirability bias or that the ward culture had become so ingrained within their way of operating that they were no longer aware of it. However, the process of carrying out observations on the wards presented several challenges which may have impacted on the data we obtained. The physical layout of most wards meant that the public spaces where the researchers spent most of their time were confined to the corridors, lounges or common rooms, dining areas and activity rooms. Patients’ bedroom or bed spaces (some of the wards had shared 4-bed bays) were respected as private. If the nurses were busy in the office there were periods where there was little ward activity and few interactions between staff and patients to observe. As with all overt observational studies, staff and patient behaviours may also have been influenced by the researcher’s presence. While some members of staff did express reservations about the observations and the researcher’s motives, researchers did observe staff engaging in socially undesirable ways suggesting that that their presence was easily forgotten by some staff. We report on reflexivity in the method section and note the influence of the background of the research team on the data collection and analysis; however, here we note the absence of nurses within the process of data collection and analysis. Nurses with experience of working on acute wards and first-hand experience within roles that were the focus of many of our observations may have offered additional insights into staff behaviour on the ward (9).

## 5 Conclusions

TULIPS had mixed success in terms of impact upon observed culture and practices across the wards studied. Barriers to making an impact on ward culture are not surprising given the power inherent within mental health care systems, with inpatient wards epitomising the extremes of this power. The power and coercive practice contrasts with policies and professional guidelines promoting co-production and collaboratively alliances with patients, as well as models of care like TULIPS, which aims to foster person-centred care and access to therapeutic interventions. The findings from this study suggest we need service leaders and a critical mass of staff on inpatient wards advocating for, and being able to deliver, more person-centred care and psychosocial interventions. In order to fulfil these roles, staff need access to training, supervision and support systems which appreciate and validate the demanding nature of working on mental health inpatient wards. On a more practical note, the layout and design of inpatient wards also needs to be addressed so nursing staff have a safe place to go to take a break from emotionally charged ward environment but the staff base on the wards is open and accessible for patients.

## Data Availability

The raw data supporting the conclusions of this article will be made available by the authors, without undue reservation.

## References

[1] NICE. Schizophrenia. In Core interventions in the treatment and management of schizophrenia in primary and secondary care (update). London: National Institute for Clinical Excellence, London (2014).

[2] Raphael J Price O Hartley S Haddock G Bucci S Berry K. Overcoming barriers to implementing ward-based psychosocial interventions in acute inpatient mental health settings: A meta-synthesis. Int J Nurs Stud. (2021) 115:103870. doi: 10.1016/j.ijnurstu.2021.103870 33486388

[3] Evlat G Wood L Glover N. A systematic review of the implementation of psychological therapies in acute mental health inpatient settings. Clin Psychol Psychothearpy. (2021) 28:1574–86. doi: 10.1002/cpp.v28.6 33870590

[4] Berry K Raphael J Wilson H Bucci S Drake RJ Edge D. A cluster randomised controlled trial of a ward-based intervention to improve access to psychologically-informed care and psychological therapy for mental health in-patients. BMC Psychiatry. (2022) 22:82. doi: 10.1186/s12888-022-03696-7 35114980 PMC8815159

[5] Bower P Gilbody S. Stepped care in psychological therapies: access, effectiveness and efficiency - Narrative literature review. Br J Psychiatry. (2005) 186:11–7. doi: 10.1192/bjp.186.1.11 15630118

[6] Berry K Handerer F Bucci S Penn G Morley H Raphael J. Ensuring that psychological interventions are delivered as intended on mental health inpatient wards. Br J Clin Psychol. (2024). doi: 10.1111/bjc.12510 PMC12057325 39467006

[7] Hong Y Mitchell SG Peterson JA Latkin. CA Tobin K Gann D. Ethnographic process evaluation: piloting an HIV prevention intervention program among injection drug users. Int J Qual Methods. (2005) 4:1–12. doi: 10.1177/160940690500400101

[8] Vindrola-Padros C Vindrola-Padros B. Quick and dirty? A systematic review of the use of rapid ethnographies in healthcare organisation and delivery. Br Med J Qual Saf. (2018) 27:321–30. doi: 10.1136/bmjqs-2017-007226 29263139

[9] Cleary M Hunt GE Horsfall J Deacon M. Ethnographic research into nursing in acute adult mental health units: A review. Issues Ment Health Nurs. (2011) 32:424–35. doi: 10.3109/01612840.2011.563339 21736465

[10] Kinderman P. A psychological model of mental disorder. Harvard Rev Psychiatry. (2015) 13:206–17. doi: 10.1080/10673220500243349 16126607

[11] Gold R. Roles in sociological observations. Soc Forces. (1958) 36:217–23. doi: 10.2307/2573808

[12] Emerson RM Fretz RI Shaw LL. Participant observation and fieldnotes. In: Atkinson P Coffey A Delamont S Lofland J Lofland L, editors. Handbook of Ethnography. Sage Publications Ltd, London (2001).

[13] Braun V Clarke V. Using thematic analysis in psychology. Qual Res Psychol. (2006) 3:77–101. doi: 10.1191/1478088706qp063oa

[14] Braun V Clarke V. Reflecting on reflexive thematic analysis, Qualitative Research in Sport. Exercise Health. (2019) 11:589–97. doi: 10.1080/2159676X.2019.1628806

[15] Alexander J Bowers L. Acute Psychiatric ward rules: a review of the literature. J Psychiatr Ment Health Nurs. (2004) 11:623–31. doi: 10.1111/j.1365-2850.2004.00770.x 15450033

[16] Doyle A Clark A. How ward rules and limit setting contribute towards restrictive practices and presentations of challenging behaviour in patients on mental health wards. Br J Ment Health Nurs. (2020) 9:2. doi: 10.12968/bjmh.2019.0008

[17] Butterworth H Wood L Rowe S. Patients’ and staff members’ experiences of restrictive practices in acute mental health in-patient settings: systematic review and thematic synthesis. Br J Open Psychiatry. (2022) 8. doi: 10.1192/bjo.2022.574 PMC9634587 36200350

[18] Lindgren B-M Ringnér A Molin J Graneheim UH. Patients’ experiences of isolation in psychiatric inpatient care – Insights from a meta-ethnographic study. Int J Ment Health Nurs. (2019) 28:7–21. doi: 10.1111/inm.2019.28.issue-1 29975446

[19] Bowers L James K Quirk A Simpson A Stewart D Hodsoll J. Reducing conflict and containment rates on acute psychiatric wards: The Safewards cluster randomised controlled trial. Int J Nurs Stud. (2015) 52:1412–22. doi: 10.1016/j.ijnurstu.2015.05.001 PMC4518134 26166187

[20] Tully SM Bucci S Berry K. My life isn’t my life, it’s the system’s”: A qualitative exploration of women’s experiences of day-to-day restrictive practices as inpatients. J Psychiatr Ment Health Nurs. (2023) 30:110–22. doi: 10.1111/jpm.12855 PMC10084424 35771190

[21] Michel L Waelli M Allen D Minvielle E. The content and meaning of administrative work: a qualitative study of nursing practices. J Advanced Nurs. (2017) 73:2023–260. doi: 10.1111/jan.2017.73.issue-9 28276090

[22] Renwick L Lavelle M Brennan G Stewart D James K Richardson M. Physical injury and workplace assault in UK mental health trusts: An analysis of formal reports. Int J Ment Health Nurs. (2016) 25:355–66. doi: 10.1111/inm.2016.25.issue-4 27170345

[23] Stewart D Bowers L. Inpatient verbal aggression: content, targets and patient characteristics. J Psychiatr Ment Health Nurs. (2012) 20:236–43. doi: 10.1111/j.1365-2850.2012.01905.x 22486899

[24] Akinola P Rayner G. Staff attitudes, beliefs and responses towards self-harm: a systematised literature review. Br J Ment Health Nurs. (2022) 11. doi: 10.12968/bjmh.2020.0006

[25] Christodoulou-Fella M Middleton N Papathanassoglou ED Karanikola MNK. Exploration of the association between nurses’ moral distress and secondary traumatic stress syndrome: implications for patient safety in mental health services. BioMed Res Int. (2017) 1908712. doi: 10.1155/2017/1908712 29209622 PMC5676344

[26] Awenat Y Peters S Shaw-Nunez E Gooding P Pratt D Haddock G. Staff experiences and perceptions of working with in-patients who are suicidal: qualitative analysis. Br J Psychiatry. (2017) 211:103–8. doi: 10.1192/bjp.bp.116.191817 PMC5537568 28642259

[27] Berry K Allsopp K Gaskin F Price O. Staff support for workplace trauma: A Freedom of Information Act request survey for NHS Trusts providing mental health care in England. J Ment Health. (2023) 7:1–5. doi: 10.1080/09638237.2023.2278094 37933756

[28] Potthoff S Kwasnicka D Avery L Finch T Gardner B Hankonen N. Changing healthcare professionals’ non-reflective processes to improve the quality of care. Soc Sci Med. (2022) 298:114840. doi: 10.1016/j.socscimed.2022.114840 35287065

[29] Bruk-Lee V Spector PE. Interpersonal conflict and stress at work: implications for employee health and well-being. In: Rossi AM Perrewé PL Meurs JA, editors. Coping and prevention. Charlotte, North Carolina: IAP Information Age Publishing (2012). p. 3–22.

[30] Mikkelsen EN Gray B Petersen A. Unconscious processes of organizing: intergroup conflict in mental health care. J Manage Stud. (2020) 57:1355–83. doi: 10.1111/joms.12611

[31] Christofides S Johnstone L Musa M. [amp]]lsquo;Chipping in’: Clinical psychologists’ descriptions of their use of formulation in multidisciplinary team working. Psychol Psychotherapy: Theory Res Pract. (2012) 85:424–35. doi: 10.1111/j.2044-8341.2011.02041.x 23080531

[32] McKenna M Brown LJ Berry K. Formulation-led care in care homes: staff perspectives on this psychological approach to managing behaviour in dementia care. Int J Older People Nurs. (2022) 17:e12465. doi: 10.1111/opn.12465 35403365 PMC9541292

[33] McKeown M Thompson G Scholes A Jones F Downe S Price O. Restraint minimisation in mental health care: legitimate or illegitimate force? ethnographic study. Sociology Health Illness. (2020) 42:449–64. doi: 10.1111/1467-9566.13015 31657030

