# Supplementary material for: Barriers to the implementation of psychosocial interventions on acute mental health wards: an ethnographic observational study
Source: Front Psychiatry. 2025 Feb 27;16:1501945. doi: 10.3389/fpsyt.2025.1501945 (PMC11920708; doi:10.3389/fpsyt.2025.1501945)
Supplement: Supplementary file 1 [file Table1.docx]

| 1. **Interventions: Motivation or interest in or positive perspectives on psychology**   Staff expressing motivation to engage with the psychologist, deliver interventions or a general positive view or interest in psychologists in general and their role on the ward. |
| --- |
| 1. **Advertising of activities: Invitation by staff**   Patients are personally invited to engage in activities by staff members. Also suggests some judgements being made about who is suitable and therefore, gatekeeping, of accessing activities. |
| 1. **Advertising of activities: Posters**   Visible posters advertising that activities are available to patients – although this didn’t always mean that they were going ahead. |
| 1. **Beliefs about patients’ suitability for the ward**   Both staff and patients express beliefs around other patients, or themselves, and if they *needed* to be on the ward, if the treatment was appropriate and/or necessary, and equally staff expressed views about patients not being appropriate for the ward due to their presentation and/or diagnosis. |
| 1. **Evidence of lacking resource: Extra resources available**   Signs that staff have extra available time or resources to engage in activities outside of their daily duties. |
| 1. **Evidence of lacking resource: Interventions are cancelled due to low staffing**   Planned activities are cancelled due to not having enough staff present on shift or an increased need meaning staff are busier than usual. |
| 1. **Evidence of lacking resource: Lacking physical resource, tools**   Insufficient resources in the form of physical resources (e.g. bedding, clothing, food, flip charts, pens etc) that prevent or act as a barrier to care, interventions or activities being completed. |
| 1. **Evidence of lacking resource: Low staffing**   Evident from staff being visibly busy and genuinely not having enough time for patients, having to wait for needs and requests being met, the presence of bank or agency staff on the ward etc |
| 1. **Evidence of lacking resource: Unexpected changes in workload**   Sudden incidents on the ward or staff being late etc can significantly impact on individuals workload and later plans for the shift. |
| 1. **Evidence of lacking resource: Perception that a lack of time is a barrier to engaging**   Expressions that staffs lack of time due to a lack of resources means they are unable to deliver/engage in study interventions. |
| 1. **Lack of meaning activity available on the ward**   There is a lack of activities being offered on the ward meaning that patients are either in their bedspaces, out on leave, or sat around in communal areas for most of the day. They are bored and do not have the opportunity to engage in meaningful (non/therapeutic activities). |
| 1. **Lack of job satisfaction - insufficient time and training to deliver care appropriately, burnout**   Frustrations around staffs job role were observed, including irritation that they were less able to engage in therapeutic work due to a lack of time and skill. There were multiple examples of burnout or stress resulting from a lack of support in their roles and individuals actively complaining of their roles or seeking to leave their job. |
| 1. **Patient to patient interventions: Patients keep to themselves.**   Patients observed keeping to themselves, not engaging with staff or patients, or avoiding engaging in in-depth interactions, spending lots of time in their bedspace and rejecting to avoid in activities. |
| 1. **Patient to patient interventions: Peer support**   Therapeutic engagements between patients including offering each other support with their anxieties and looking out for one and other. |
| 1. **Positivity about job role**   Staff express positivity about their roles, show signs of enjoying the work. They are enthusiastic and passionate about their positions and feeling value in helping others through their work. |
| 1. **Interventions: Consideration of reflection on psychology or motivation behind behaviour**   Evidence of staff considering how individuals experiences and psychology influences the behaviour being presented, during ward round, formulation sessions, supervision, generally in discussion with other staff. This may be an outcome of the intervention, if not seen at TP1. |
| 1. **Presence, visibility of staff: Office door**   The office door is often shut by staff to close themselves into the nursing office and away from patients. In other instances, staff keep the door open or ajar so that patients may freely interact with them or make requests. |
| 1. **Staff and patient interactions: Examples of untherapeutic practice**   Staff are uncompassionate with patients. This includes being condescending or talking to patients like they are children, neglecting patients. Disregarding their needs or concerns or do not take the time to ease their concerns, brushing them off. Restrictive practices are used, or staff can be rude, or even actively bully staff. |
| 1. **Staff and patient interactions: Conflict**   Conflict between staff and patients, this is often related to disagreement over medication, needs being neglected or not being met, or having to wait long periods before requests were met, also miscommunication and disorganisation within the staff team. False advertisement of activities and lack of staff time. |
| 1. **Interventions: Patients are not in interested or anxious of TP, Interventions, Study**   Patients do not want to engage with the trial psychologist or research activities or may express anxieties around doing so. |
| 1. **Staff and patient interactions: Not offering choice or flexibility**   Staff were strict and inflexible when engaging with patients, making decisions, or facilitating care. Patients would often make request around leave or treatment. |
| 1. **Interventions: Awareness, Knowledge of TP, Intervention**   Awareness varied, some were aware of the trial psychologists presence on wards but not aware of the wider study. There was often confusion with staff or patients thinking the observer was a psychologist. Sometimes staff were aware of the study but didn’t have a knowledge of what this involved. Many staff and patients were not aware of the interventions aiming to be delivered (typically due to having not been offered them). This tended to vary between wards based on how present and integrated the trial psychologist was. |
| 1. **Staff and patient interactions: Staff engage positively, genuinely with patients**   Observed signs of positive relationships between staff and patients. Normalised interactions and conversations putting them on a more similar standing, seeking out interactions, laughing and joking |
| 1. **Staff and patient interactions: Staff engage superficially with patients**   Tick box interactions that are task focused, for example during medication administration, or the completion of observations. Staff use these as an opportunity to engage in short interactions with patients but these are without any depth. |
| 1. **Staff and patient interactions: Staff escalate or cause conflict**   Staff attempts to de-escalate conflict take the wrong turn, alternatively, staff directly cause conflict or behave inappropriate in response to conflict from patients (such as shouting back or squaring up) |
| 1. **Staff and patient interactions: Staff ignore or avoid patients**   Staff deflect requests from patients, re-directing them to other staff members or simply saying they cannot complete these requests at this time. They directly ignore patients approaching them or patient behaviour, which is inappropriate or upsetting to others, likely in a bid to not cause conflict, they also avoid engaging with patients. |
| 1. **Staff and patient interactions: Staff Knowledge, Skills**   Staff’s knowledge and skills varied. Often staff did not have answers for patients’ questions or were unclear on what services were available to them. They also showed a lack of skill in providing appropriate support at times. |
| 1. **Staff and patient interactions: Staff knowledge of patients**   This varied significantly. Staff who spent more time with patients had a greater knowledge of their histories, experiences, and the meaning behind their behaviour. Whilst others knowledge was based solely on notes. |
| 1. **Staff and patient interactions: Staff provide or offer in-depth support or engage compassionately**   Staff offer support to patients who seem upset or struggling, they may engage in one-to-one interventions or simply listen and provide encouragement and compassionate engagement (such as hands on shoulders, verbal encouragement, and reassurance). |
| 1. **Staff and patient interactions: Staff regard for or belief of or bias towards patients**   Staff presented a lack of regard or belief of patient’s opinions, views or complains. Some staff presented bias towards specific patients sometimes based on the diagnosis they were labelled with. |
| 1. **Staff and patient interactions: Staff reliance on medication**   Over and another therapeutic support staff rely on medication and PRN when patients are presenting as distressed or in need of support. |
| 1. **Staff and patient interactions: Staff take time to explain**   They take the time to explain decision making, what medication they are prescribed and what it involved, or decisions that are made around care. |
| 1. **Staff and patient interactions: Transparency between staff and patients**   Honesty around decision making, such as “*we’ve got no staff to take you out”* in places of evading explanations or reasoning. |
| 1. **Staff to staff interactions: Contradictions or inconsistencies in care**   Staff practice often contradicted each other create inconsistent treat or decisions around care and sometimes leading to conflict between staff and patients, or between staff members. |
| 1. **Staff to staff interactions: Conflict between staff**   Conflict between staff groups was common such as staff’s frustration at doctors’ behaviour, decisions or lateness. There was also conflict with decisions made by management. Tension between HCAs and nurses was sometimes apparent or conflict between individual staff members who did not get along. |
| 1. **Staff to staff interactions: Evidence of hierarchy between staff**   Hierarchies existed within the staff team both pre-defined hierarchies between those in senior and junior positions. But also, less prescribed hierarchies identified by who felt comfortable to engage in meetings or lead discussions. |
| 1. **Staff to staff interactions: Examples of peer support**   Visible comradery between staff and examples of them providing support to one and other, checking on each other’s wellbeing after incidents and generally support one and other. This was visible within staff groups such as bank and agency supporting one and other. |
| 1. **Staff to staff interactions: Hostility to Outsiders**   Staff are hostile, rude, or isolate outsiders such as the researchers or bank/agency staff. Sometimes this included staff who did not work on the ward or were not perceived as the part of the team, the ward but were not senior, such as cleaners, chaplains. |
| 1. **Staff to staff interactions: Informal relationships**   Personal and informal relationships between staff members, visible through social interactions and engagements whilst on the ward or discussing personal lives. |
| 1. **Staff to staff interactions: Poor communication and organisation between staff**   Lack of communication between staff members sometimes resulting in care and needs not being met. There is also a lack of communication from staff to patients and carers. |
| 1. **Staff to staff interactions: Professionalism**   Professionalism varied between staff with some staff seen gossiping about others in the presence of patients and other staff members. Visible tension on the wards between staff members. Others remained professional in the place of conflict in their roles. |
| 1. **Staff to staff interactions: Teamwork**   Staff teaming together to deliver care, such as psychiatrists and the nursing team working together to deliver care (As opposed to the psychiatrist dictating to staff how treatment should be delivered). They may work together during meetings such as formulations or ward round. |
| 1. **Staff and patient interactions: Boundaries**   Staff set or reinstate boundaries with patients around relationships, language, or access to the office. These boundaries vary between staff members with some being comparably un-boundaries. |
| 1. **Staff and patient interactions: Patients are sceptical of staff**   Patients are disbelieving of staff attempts to offer support or engage with them. They may act in a way that suggests they are nervous or distrustful of staff motives. |
| 1. **TP Presence and engagement: Staff and TP get on**   Evidence of informal relationships, seen as the part of the nursing team. The staff are relaxed around them and positive about the TPs presence. |
| 1. **Staff and patient interactions: Social distance between staff and patients**   Visible distance between staff and patients. For example eating at separate tables, signs of hierarchy and distance between the staff and patients. |
| 1. **TP Presence and engagement: TP engages with patients**   In the delivery of in-depth interventions and one-to-ones, offering emotional support or listening to their concerns. |
| 1. **TP Presence and engagement: TP engages with patients Superficially.**   The TP is present on the ward but engages only superficially with the patients such as asking briefly how they are, greeting them, smiling etc |
| 1. **TP Presence and engagement: TP supports staff informally.**   Whilst present on the ward the TP provides support to the staff, such as informal formulations and supervisions in the nursing office and providing expertise during discussions with staff that were not planned. |
| 1. **Ward environment: Patients have a positive view of the ward**   Patients believe the ward is positive, the staff are helpful and caring. |

| 1. **Patient to patient interventions: Positive patient interaction**   Positive engagements between patients, such as laughing joking engaging in activities together. |
| --- |
| 1. **Patient to patient interventions: Conflict**   Conflict between patients, some appear to seek out conflict with others or actively “wind each other up”. |
| 1. **Interventions: Staff Scepticism in the project**   Disbelief in its effectiveness and/or suitability for the ward and/or the trial psychologist themselves |
| 1. **Interventions: Who led interventions**   Occupational therapist (Ward 4), Assistant psychologist and Occupational therapist (Ward 5) |
| 1. **Interventions: Intervention suitability**   Considerations or decisions or discussion around if patients are suitable for specific interventions. |
| 1. **Interventions: Interventions communicated in Ward round/Handover**   Mention of the studies interventions by staff in ward round and handovers. |
| 1. **Evidence of revolving door**   (multiple) Readmissions to the wards |
| 1. **Hawthorne Effect**   Evidence of staff or patients changing their behaviour due to the presence of the researcher |
| 1. **Activities are available on the ward**   This includes study related and unrelated activities. |
| 1. **Evidence of lacking resource: Not enough beds** |
| 1. **Interventions: Staff are not implementing interventions** |
| 1. **Evidence of lacking resource: Staff respond to requests immediately** |
| 1. **Interventions: Patients do not feel comfortable being observed, interviewed** |
| 1. **Interventions: Staff do not reflect on patients psychology** |
| 1. **Presence, visibility of staff: Patients congregate at the office door**   Patients congregate around the office door waiting for staff to facilitate requests as they are not present on ward. |
| 1. **Presence, visibility of staff: Staff are in the office**   Staff are closed in the office often working on computers or chatting with peers. |
| 1. **Presence, visibility of staff: Staff are not present on the ward**   There are none, or minimal staff present in communal spaces on the ward. |
| 1. **Presence, visibility of staff: Staff are not present on the ward (engaging with patients)**   Staff are present in communal spaces and engaging with patients who are present. |
| 1. **Presence, visibility of staff: Staff are not present on the ward (not engaging with patients)**   Staff are present in communal spaces, alongside patients but do not engage with them. |
| 1. **Staff and patient interactions: Accessing advocacy.**   Staff support patients to contact their advocate. |
| 1. **Staff and patient interactions: Patients avoid or ignore staff or try not to cause bother**   Patients keep to themselves around staff, not seeking out support or wanting to bring attention to themselves |
| 1. **Staff and patient interactions: Patients seek support** |
| 1. **Staff and patient interactions: Some patients take up more staff time** |
| 1. **Staff and patient interactions: Some staff are more compassionate or caring than others** |
| 1. **Staff and patient interactions: Staff de-escalate conflict** |
| 1. **Staff and patient interactions: Staff going out of their way for patients**   Going the extra mile outside of job expectations for patients. |
| 1. **Staff and patient interactions: Staff offering choice or flexibility or least restrictive practices.** |
| 1. **Staff to staff interactions: Anxieties around external scrutiny**   Staff are nervous of scrutiny from those outside of the ward on their practice or behaviour. |
| 1. **Staff to staff interactions: Lack of support between staff**   Staff do not support one and other, there is a lack of comradery of even structured support after incidents occur. |
| 1. **Staff to staff interactions: Staff discussing patients: Complaints or Gossip** |
| 1. **Staff to staff interactions: Staff discussing patients: Discussing care or wellbeing.** |
| 1. **Staff to staff interactions: Staff discussing patients: Positive or fondly.** |
| 1. **Staff to staff interactions: Supporting outsiders.**   Staff support or are kind to outsiders. |
| 1. **TP Presence and engagement: TP does not engage**   TP is present on the ward but does not engage with staff or patients while visible. |
| 1. **TP Presence and engagement: TP has rapport, relationship with patients** |
| 1. **TP Presence and engagement: TP is present on the ward** |
| 1. **TP Presence and engagement: TP supports staff formally**   Through attendance in formulations and supervisions sessions led by the Trial psychologist. |
| 1. **TP Presence and engagement: TPs office is on the ward** |
| 1. **Ward round is delayed or cancelled or staff are late to handover or ward round** |
| 1. **Ward environment: Lack of suitable space**   To deliver interventions |
| 1. **Ward environment: Negative outcomes of the ward**   the untherapeutic environment, the chaos or restrictions resulting in negative outcomes for patients or conflict. |
| 1. **Ward environment: Patients have a negative view of the ward**   They are frustrated with the ward and do not view them as therapeutic or supportive. |
| 1. **Ward environment: Ward is Calm** |
| 1. **Ward environment: Ward is Chaotic** |
| 1. **Ward environment: Ward is empty** |
| 1. **Ward environment: Ward is restrictive** |
